# Supplementary material for: Molecular architecture of the luminal ring of the Xenopus laevis nuclear pore complex
Source: Cell Res. 2020 May 4;30(6):532–40. doi: 10.1038/s41422-020-0320-y (PMC7264284; doi:10.1038/s41422-020-0320-y)
Supplement: Supplementary file 1 — Supplementary Figure S1 [file 41422_2020_320_MOESM1_ESM.pdf]

Supplementary information, Fig. S1

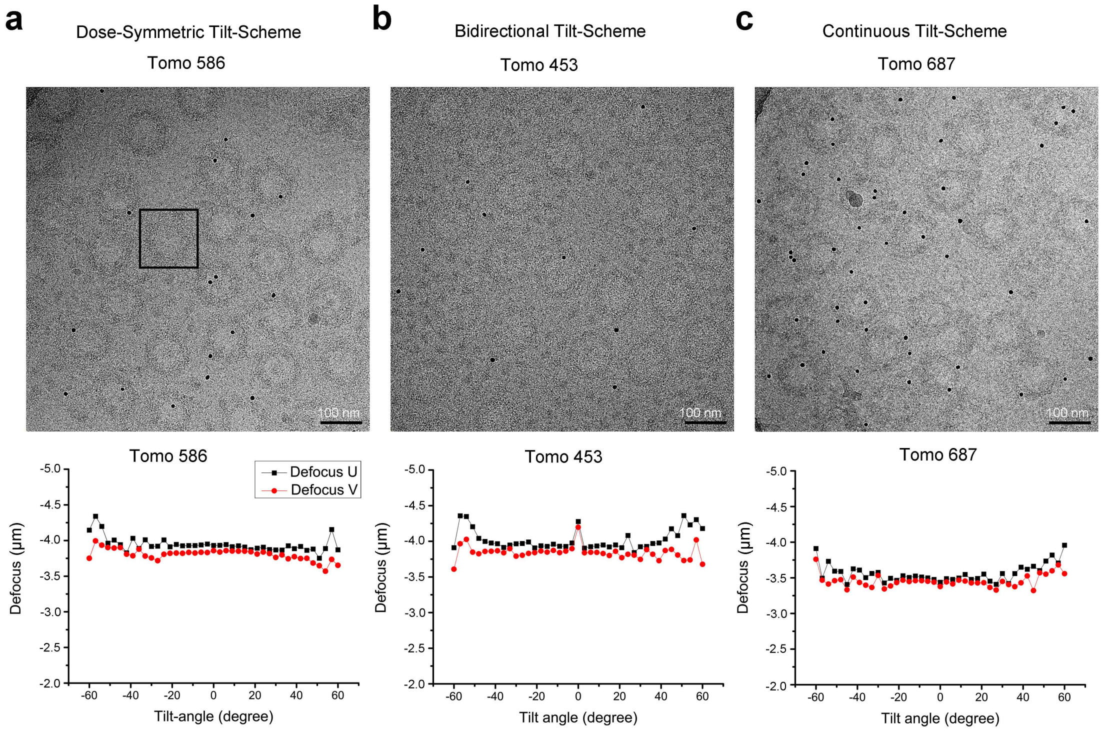

Supplementary information, Fig. S1 | Representative 0°-tilt micrographs and the associated defocus values for three tilt-schemes used in this study. **a**, Dose-symmetric tilt-scheme (-60° to 60°). 240 tilt-series were recorded. Shown here (tomo 586) is a representative example. **b**, Bidirectional tilt-scheme (0° to -60° and 3° to 60°). 874 tilt-series were recorded. Shown here (tomo 453) is a representative example. **c**, Continuous tilt-scheme (-60° to 60°). 311 tilt-series were recorded. Shown here (tomo 687) is a representative example. In each of the three cases, the 0°-tilt micrograph and the defocus value for the specified tilt-series are shown in the upper and lower panels, respectively.
