# Supplementary material for: Molecular architecture of the luminal ring of the Xenopus laevis nuclear pore complex
Source: Cell Res. 2020 May 4;30(6):532–40. doi: 10.1038/s41422-020-0320-y (PMC7264284; doi:10.1038/s41422-020-0320-y)
Supplement: Supplementary file 2 — Supplementary Figure S2 [file 41422_2020_320_MOESM2_ESM.pdf]

Supplementary information, Fig. S2

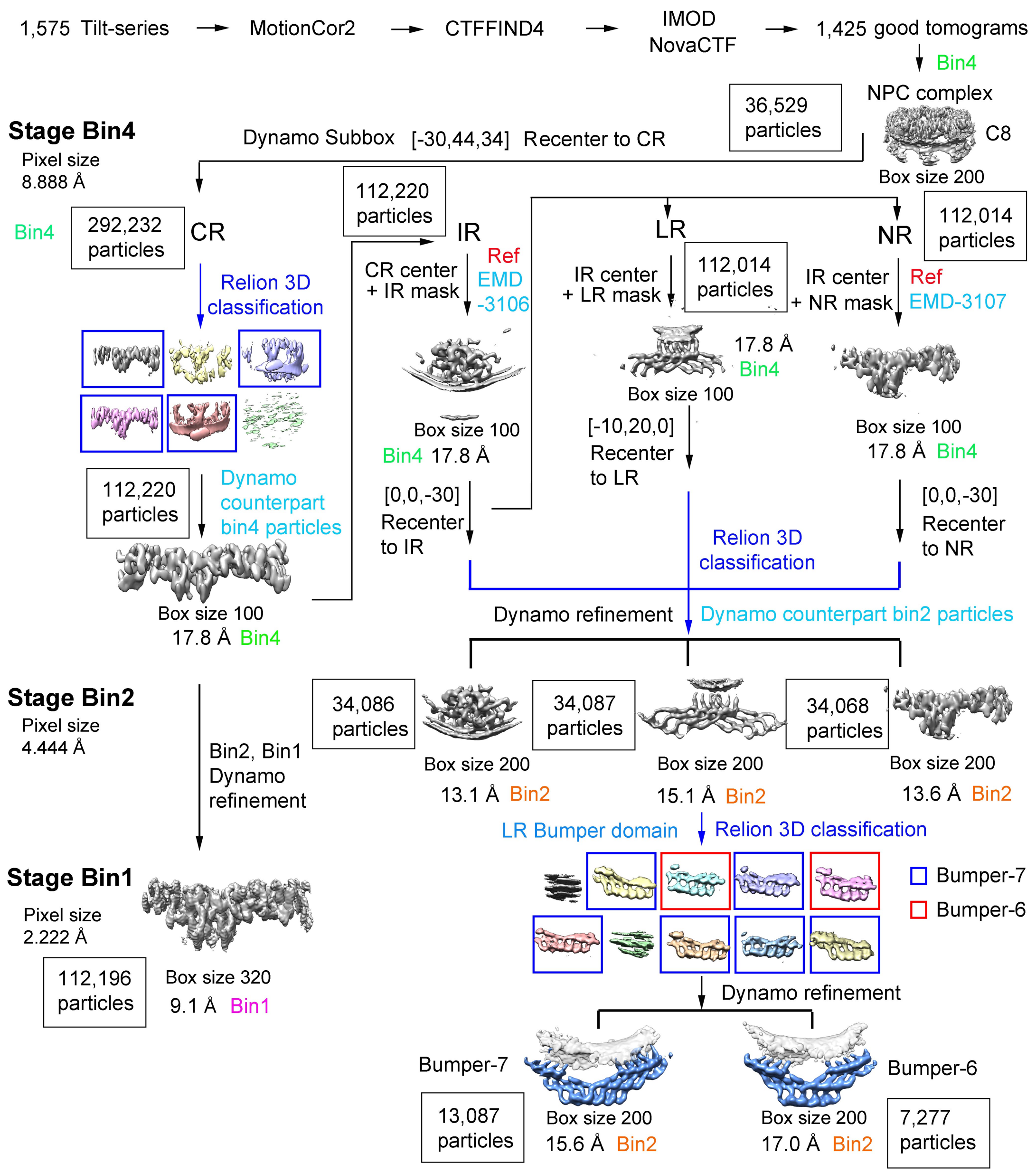

Supplementary information, Fig. S2 | A processing flow chart of the cryo-ET data on the NPC from *Xenopus laevis* (*X. laevis*). For detailed description, please refer to the Methods. CR, cytoplasmic ring; IR, inner ring; LR, luminal ring; NR, nuclear ring.
