# Supplementary material for: Molecular architecture of the luminal ring of the Xenopus laevis nuclear pore complex
Source: Cell Res. 2020 May 4;30(6):532–40. doi: 10.1038/s41422-020-0320-y (PMC7264284; doi:10.1038/s41422-020-0320-y)
Supplement: Supplementary file 3 — Supplementary Figure S3 [file 41422_2020_320_MOESM3_ESM.pdf]

Supplementary information, Fig. S3

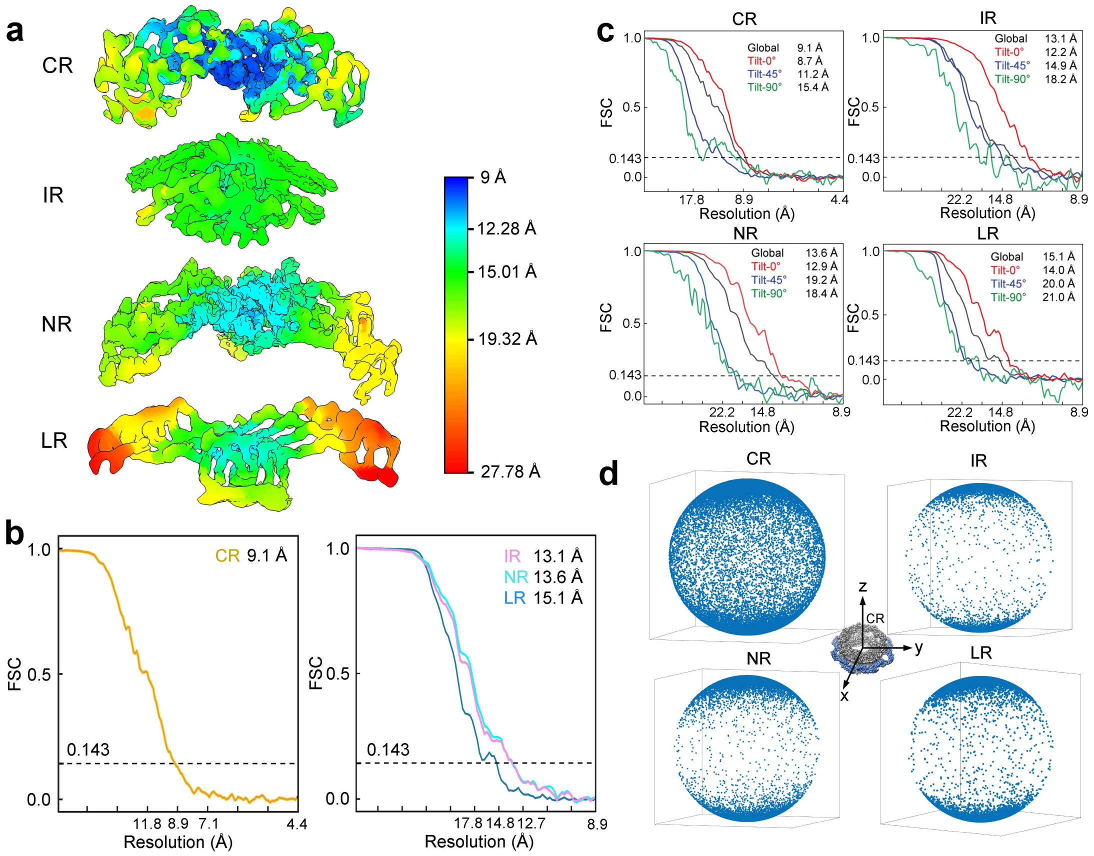

Supplementary information, Fig. S3 | Average resolution and anisotropy of the cryo-ET reconstructions for the NPC from *X. laevis*. **a**, The STA-based cryo-ET reconstructions of the CR, IR, NR, and LR subunits. The resolution range for each of these reconstructions is color-coded. **b**, The Fourier shell correlation (FSC) curves for the reconstructions of the CR, IR, NR, and LR subunits. On the basis of the FSC criterion of 0.143, the reconstructions of the CR, IR, NR, and LR subunits have average resolutions of 9.1 Å, 13.1 Å, 13.6 Å, and 15.1 Å, respectively. **c**, The cryo-ET reconstruction is anisotropic. Shown here are the Fourier shell correlation (FSC) curves of the CR, IR, NR and LR subunits in three different orientations. The FSC curves for tilt 0°, 45°, and 90° (named Tilt-0°, Tilt-45° and Tilt-90°) were calculated with cone masks in Fourier space and colored red, blue, and green, respectively. As a reference, the FSC curve for the global CR subunit is shown and colored black. The resolutions were calculated on the basis of the FSC criterion of 0.143. **d**, Angular distribution of particles for reconstruction of the CR, IR, NR and LR subunits.
