# Supplementary material for: Molecular architecture of the luminal ring of the Xenopus laevis nuclear pore complex
Source: Cell Res. 2020 May 4;30(6):532–40. doi: 10.1038/s41422-020-0320-y (PMC7264284; doi:10.1038/s41422-020-0320-y)
Supplement: Supplementary file 4 — Supplementary Figure S4 [file 41422_2020_320_MOESM4_ESM.pdf]

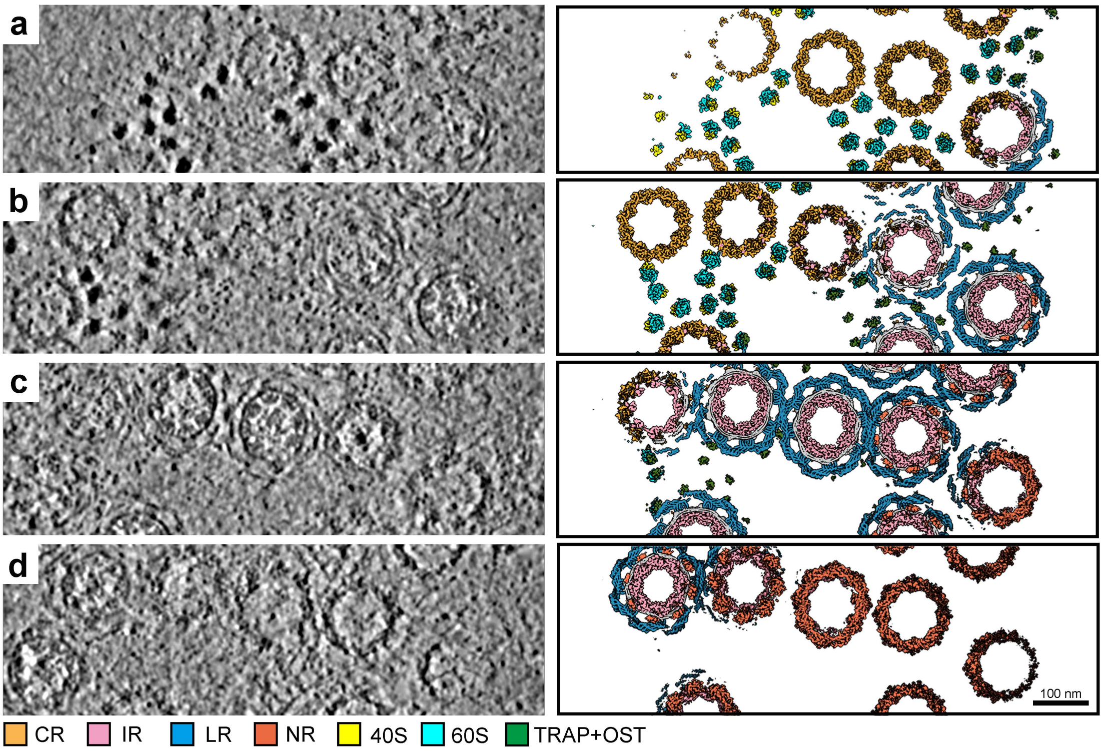

Supplementary information, Fig. S4 | Three-dimensional organization of the NPC particles in a local region of the *X. laevis* NE. Four consecutive tomographic 2D slices are displayed in the left columns of panels **a** through **d**, with panel **a** on the cytoplasmic side and panel **d** on the nucleoplasmic side. These slices, each measuring 8.89 Å in thickness, are evenly spaced with a separation of 17.78 nm between two neighbouring slices. Structural interpretation of the regions is shown in the right columns of panels **a** through **d**, where the reconstructions for the NPC subunits and ribosomes were back-projected onto the original tomograms based on the refined coordinates of the individual particles. Ribosomes: 40S: Small ribosome subunit; 60S: Large ribosome subunit; TRAP: translocon-associated protein complex; OST: oligosaccharyl transferase.
