# Supplementary material for: Molecular architecture of the luminal ring of the Xenopus laevis nuclear pore complex
Source: Cell Res. 2020 May 4;30(6):532–40. doi: 10.1038/s41422-020-0320-y (PMC7264284; doi:10.1038/s41422-020-0320-y)
Supplement: Supplementary file 5 — Supplementary Figure S5 [file 41422_2020_320_MOESM5_ESM.pdf]

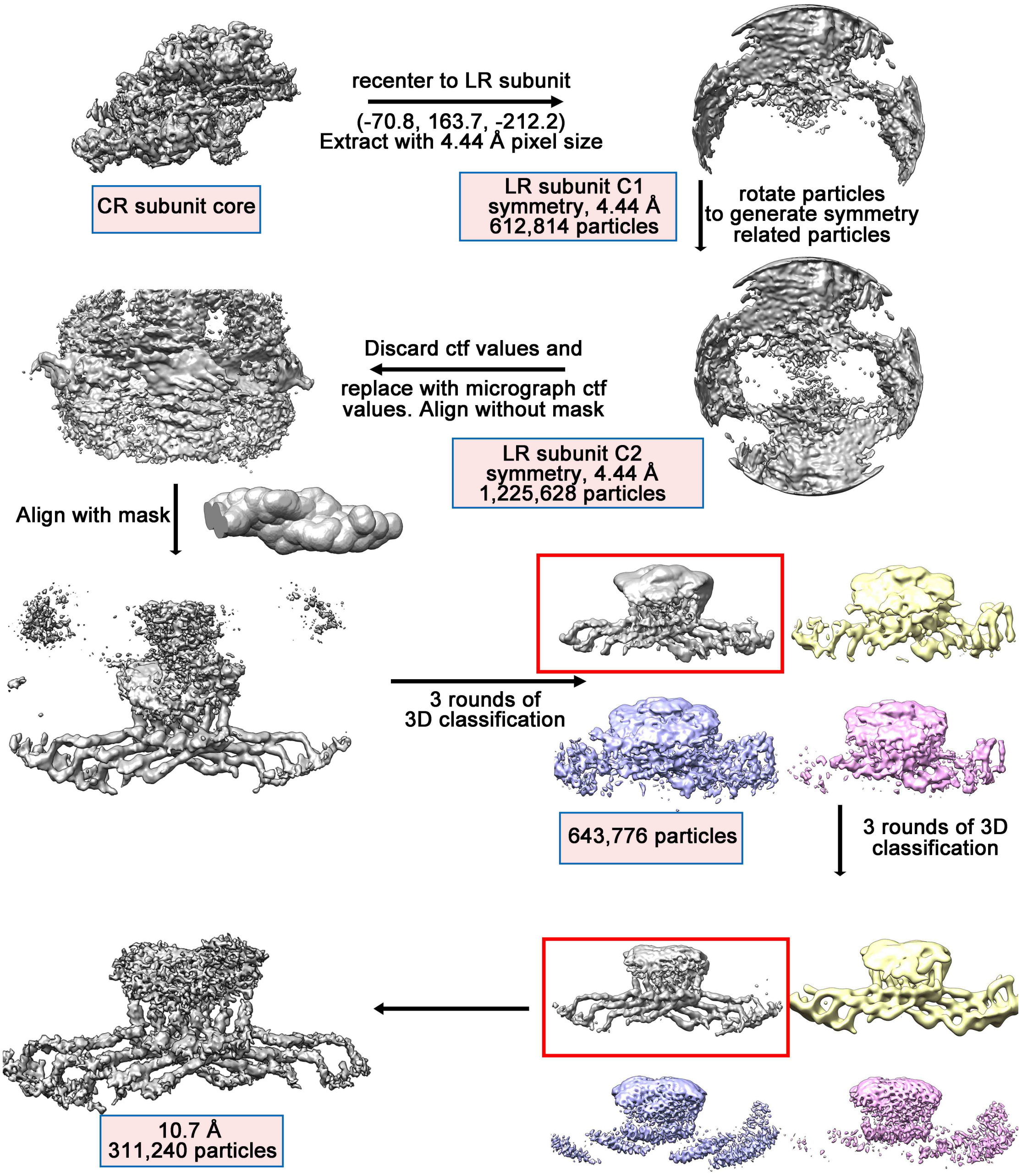

Supplementary information, Fig. S5 | A flow chart for the processing of the cryo-EM data on the LR subunit of the NPC from *X. laevis*. Single-particle analysis (SPA) was applied in data processing. For detailed description, please refer to the Methods.
