# Supplementary material for: Molecular architecture of the luminal ring of the Xenopus laevis nuclear pore complex
Source: Cell Res. 2020 May 4;30(6):532–40. doi: 10.1038/s41422-020-0320-y (PMC7264284; doi:10.1038/s41422-020-0320-y)
Supplement: Supplementary file 6 — Supplementary Figure S6 [file 41422_2020_320_MOESM6_ESM.pdf]

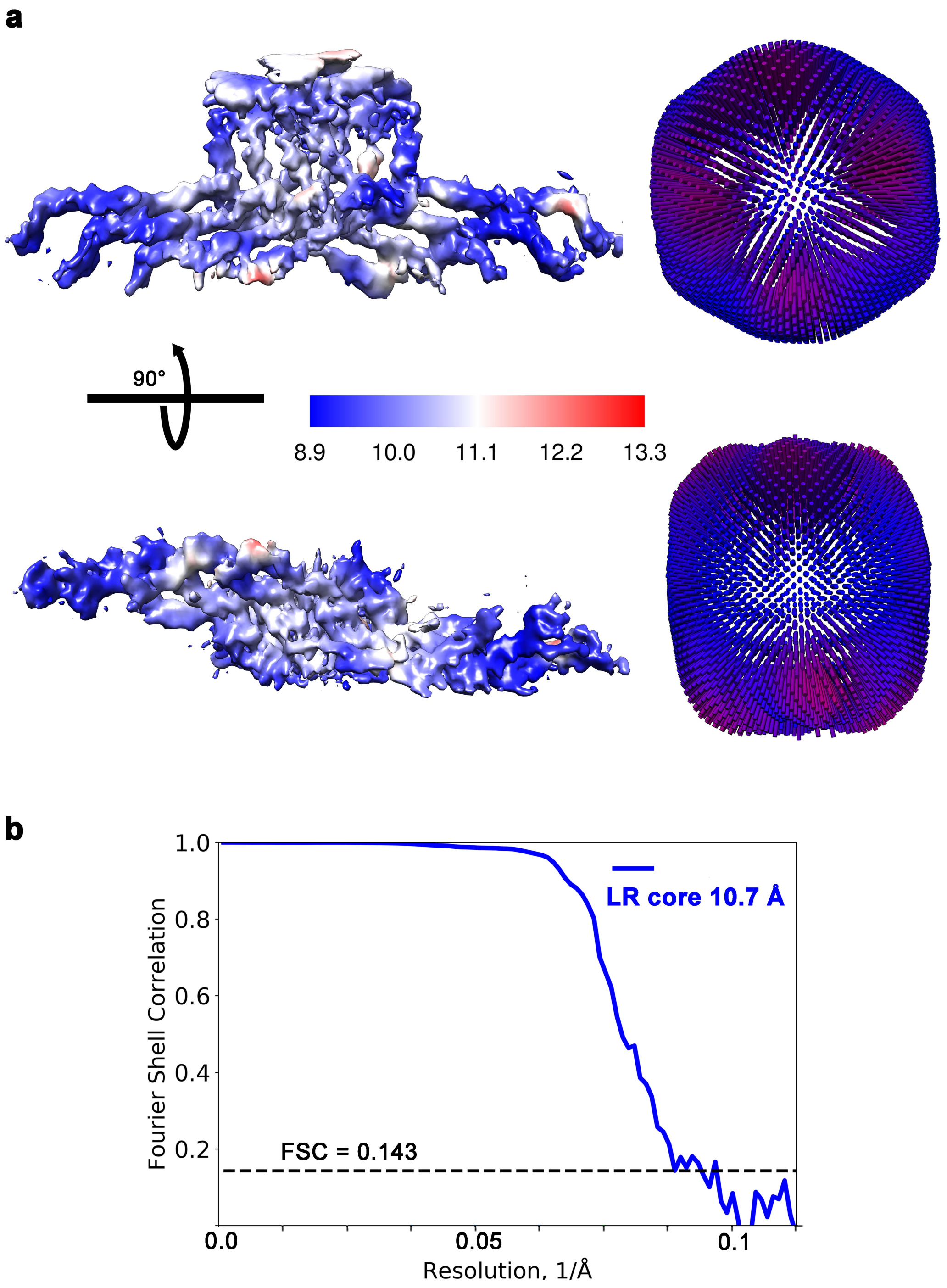

Supplementary information, Fig. S6 | Reconstruction of the LR subunit of the *X. laevis* NPC by the SPA-based cryo-EM approach. **a**, The SPA cryo-EM reconstruction of the LR subunit. The resolution range is color-coded. Two mutually perpendicular views are shown. The corresponding cylinder representations of angular distribution are shown on the right. **b**, The FSC curve for the SPA-based

reconstruction of the LR subunit. On the basis of the FSC criterion of 0.143, the reconstruction of the LR subunit has an average resolution of 10.7 Å.
