# Supplementary material for: Molecular architecture of the luminal ring of the Xenopus laevis nuclear pore complex
Source: Cell Res. 2020 May 4;30(6):532–40. doi: 10.1038/s41422-020-0320-y (PMC7264284; doi:10.1038/s41422-020-0320-y)
Supplement: Supplementary file 7 — Supplementary Figure S7 [file 41422_2020_320_MOESM7_ESM.pdf]

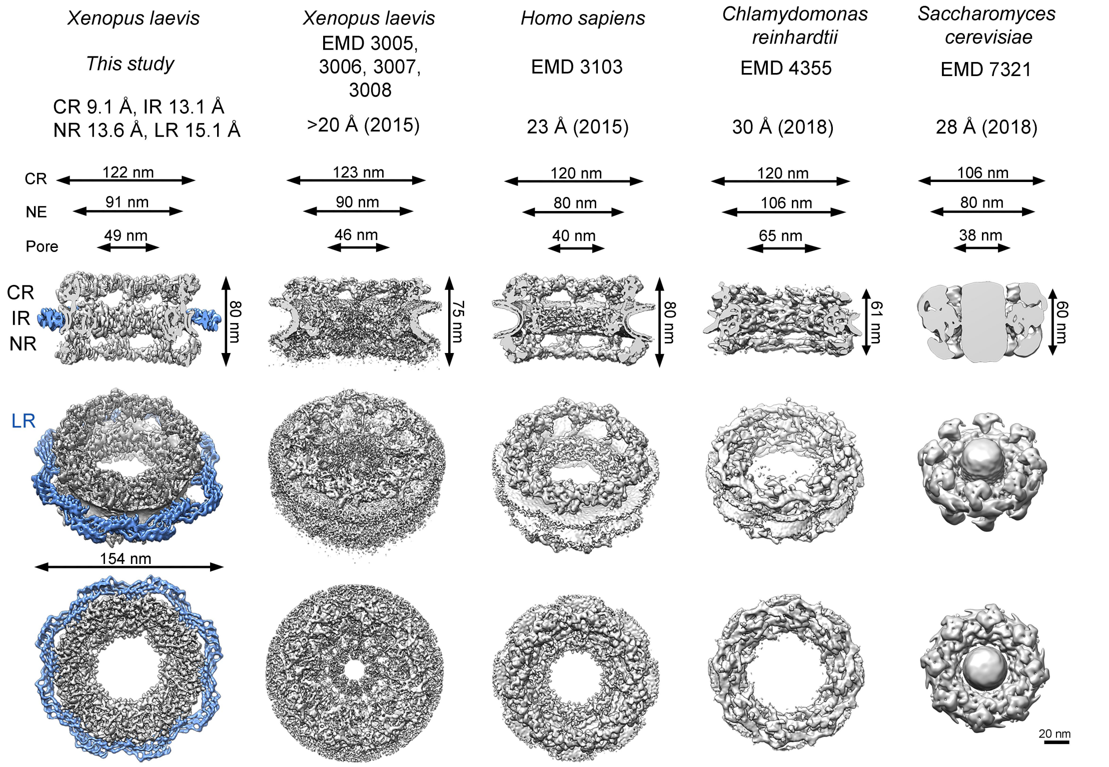

Supplementary information, Fig. S7 | Structural comparison of the cryo-ET reconstructions of the NPC. Surface representations of cryo-ET reconstructions are shown to scale for *X. laevis* (this study), *X. laevis* (accession code EMD 3005-3008)[1], *Homo sapiens* (EMD 3103)[2], *Chlamydomonas reinhardtii* (EMD 4355)[3] and *Saccharomyces cerevisiae* (EMD 7321)[4]. Three views of each reconstruction are shown: cross-section side view, 45°-tilt view, and top view from cytoplasmic side. The LR observed in this study is highlighted in marine.

References

1 Eibauer, M. *et al*. Structure and gating of the nuclear pore complex. *Nat Commun* **6**, 7532, doi:10.1038/ncomms8532 (2015).

2 von Appen, A. *et al*. In situ structural analysis of the human nuclear pore complex. *Nature* **526**, 140-143, doi:10.1038/nature15381 (2015).

3 Mosalaganti, S. *et al*. In situ architecture of the algal nuclear pore complex. *Nat Commun* **9**, 2361, doi:10.1038/s41467-018-04739-y (2018).

4 Kim, S. J. *et al*. Integrative structure and functional anatomy of a nuclear pore complex. *Nature* **555**, 475-482, doi:10.1038/nature26003 (2018).
