# Supplementary material for: Molecular architecture of the luminal ring of the Xenopus laevis nuclear pore complex
Source: Cell Res. 2020 May 4;30(6):532–40. doi: 10.1038/s41422-020-0320-y (PMC7264284; doi:10.1038/s41422-020-0320-y)
Supplement: Supplementary file 8 — Supplementary Figure S8 [file 41422_2020_320_MOESM8_ESM.pdf]

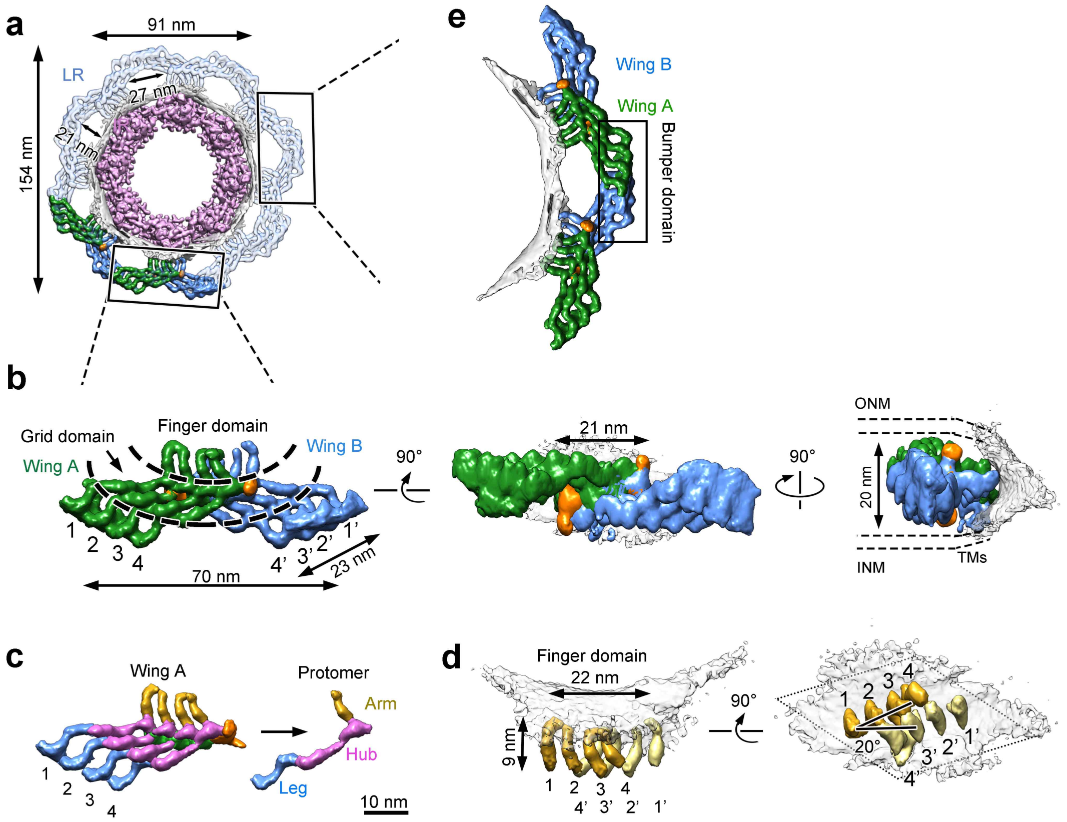

Supplementary information, Fig. S8 | Structure of the LR subunit by the STA approach. **a**, Eight LR subunits form a circular scaffold around the fusion between the inner (INM) and outer nuclear membranes (ONM). The NPC is viewed along the nucleocytoplasmic axis. Two complete LR subunits are highlighted. Within each LR subunit, two wings are colored green and marine. **b**, Structure of the LR subunit. Each butterfly-shaped LR subunit comprises two symmetric wings: Wing-A (green) and Wing-B (blue), which interact with each other through an extended interface (orange). The LR subunit has a Finger domain and a Grid domain. Three mutually perpendicular views are shown. **c**, Each wing of the LR subunit comprises four planar, elongated, tubular protomers (numbered 1 through 4). Each protomer contains an arm (colored gold), a hub (magenta), and a leg (blue). The four protomers in each wing form two

pairs (1-2 and 3-4), with the arms of each pair connected to each other through their tips. Scale bar, 10 nm. **d**, The Finger domain directly contacts the fusion of nuclear membranes. **e**, The Bumper domain is formed between two meighboring LR subunits. Four legs from Wing-A of an LR subunit interact with four legs form Wing-B of the neighboring LR subunit to form the Bumper domain.
