# Supplementary material for: Molecular architecture of the luminal ring of the Xenopus laevis nuclear pore complex
Source: Cell Res. 2020 May 4;30(6):532–40. doi: 10.1038/s41422-020-0320-y (PMC7264284; doi:10.1038/s41422-020-0320-y)
Supplement: Supplementary file 9 — Supplementary Figure S9 [file 41422_2020_320_MOESM9_ESM.pdf]

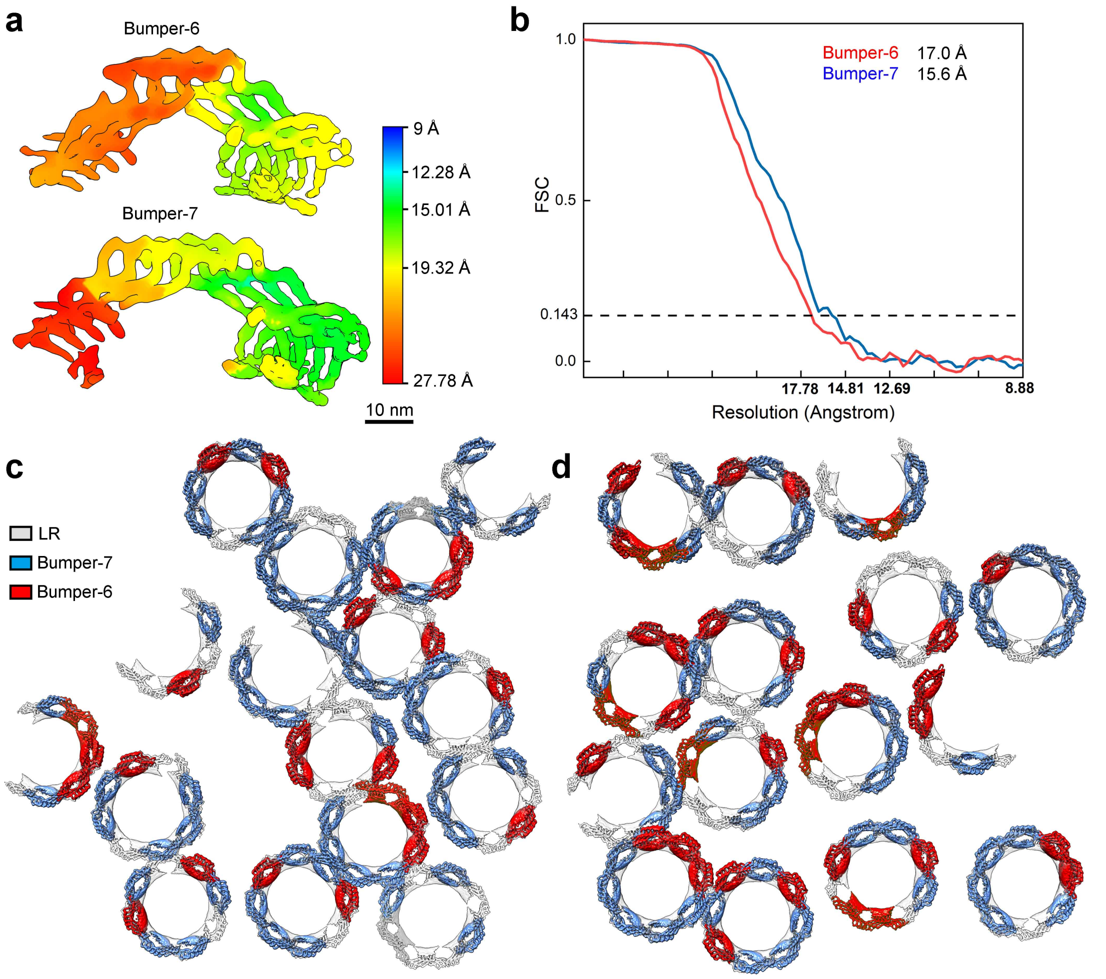

Supplementary information, Fig. S9 | Cryo-ET analysis of Bumper-6 and Bumper-7. **a**, The local resolutions for Bumper-6 and Bumper-7 are color-coded in the cryo-ET reconstructions. Scale bar, 10 nm. **b**, The FSC curves for Bumper-6 (red) and Bumper-7 (blue). The resolutions for Bumper-6 and -7 are estimated to be 17.0 Å and 15.6 Å, respectively, on the basis of the FSC criterion of 0.143. **c**, A local region of the tomogram with the refined NPC particles. The reconstructions for Bumper-7 (marine), Bumper-6 (red), and the LR subunit (grey) were back-projected onto the original tomograms based on the refined coordinates of the individual particles. **d**, Another local region of the tomogram with the refined NPC particles. Coloring scheme is the same as in panel **c**.
