# Supplementary material for: Molecular architecture of the luminal ring of the Xenopus laevis nuclear pore complex
Source: Cell Res. 2020 May 4;30(6):532–40. doi: 10.1038/s41422-020-0320-y (PMC7264284; doi:10.1038/s41422-020-0320-y)
Supplement: Supplementary file 10 — Supplementary Figure S10 [file 41422_2020_320_MOESM10_ESM.pdf]

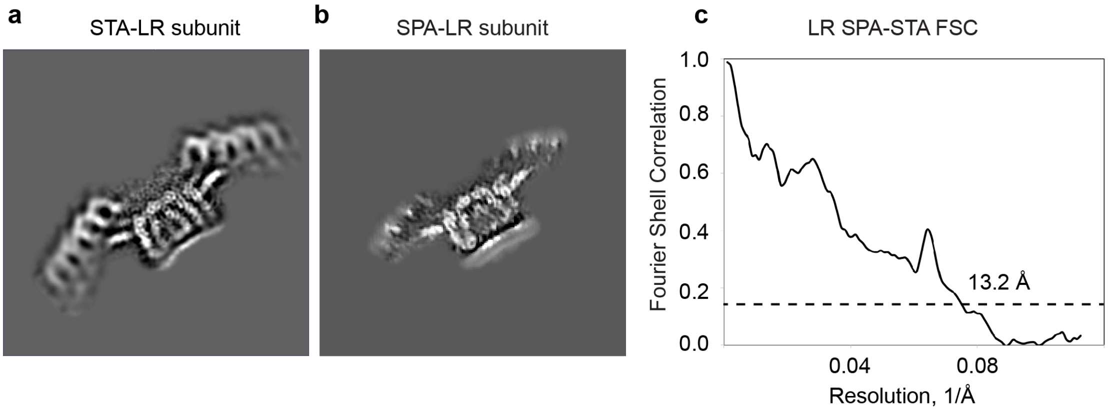

Supplementary information, Fig. S10 | Comparison of the STA and SPA reconstruction of the LR subunit. **a**, A central slice of the LR reconstruction by the STA approach. The average local resolution in the Finger and Grid domains is 12.6 Å. **b**, A central slice of the LR reconstruction by the SPA approach. For both the STA and SPA reconstructions, similar features are observed for the Finger and the Grid domains. In contrast to STA-based reconstruction, the two Bumper domains are missing in the SPA-based reconstruction, due to application of a considerably smaller alignment mask. **c**, The FSC curve of the SPA reconstruction with the STA reconstruction shows agreement of up to 13.2 Å based on the FSC criterion of 0.143. A common mask was used.
