# Supplementary material for: Molecular architecture of the luminal ring of the Xenopus laevis nuclear pore complex
Source: Cell Res. 2020 May 4;30(6):532–40. doi: 10.1038/s41422-020-0320-y (PMC7264284; doi:10.1038/s41422-020-0320-y)
Supplement: Supplementary file 11 — Supplementary Figure S11 [file 41422_2020_320_MOESM11_ESM.pdf]

## Supplementary information, Fig. S11

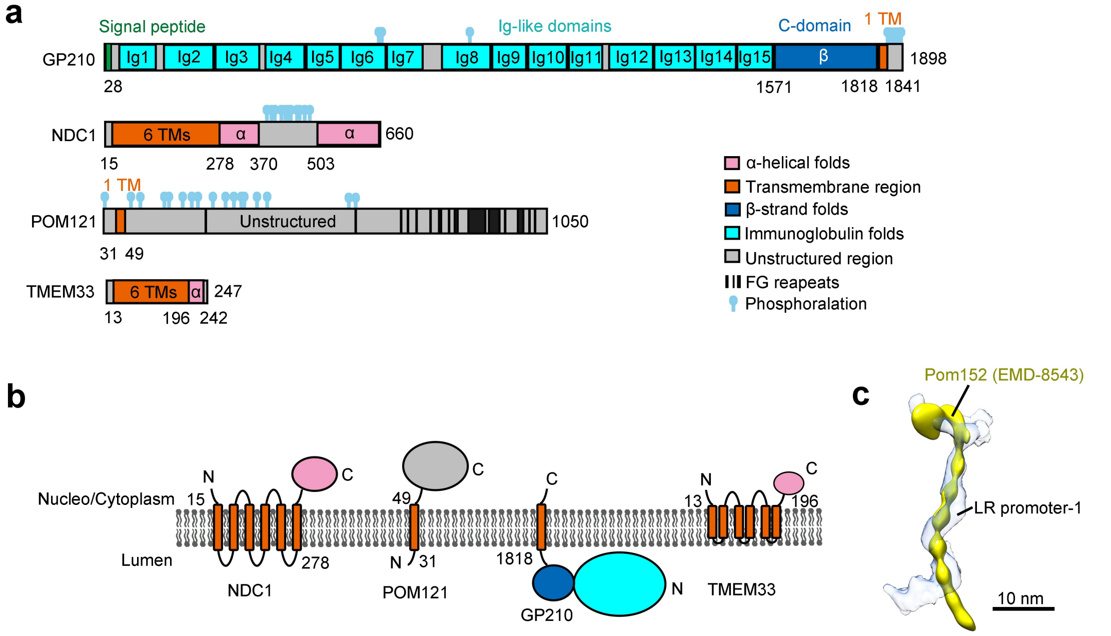


**Supplementary information, Fig. S11 | Domain structure of four candidate integral membrane proteins in the LR.** **a**, A schematic diagram of the four candidate protein components of the vertebrate LR. The four proteins and the domains within each protein are drawn to scale. GP210 (Pom152 in yeast) is the largest of the three proteins, with 1898 amino acids. GP210 sequentially contains 15 predicted Ig-like domain, a C-domain rich in β-strands, a TM, and about 57 residues at the C-terminus. **b**, A topology diagram of the four candidate protein components of the LR. For GP210, all sequences N-terminal to the TM are in the lumen of the NE. In contrast, for NDC1, only the sequences connecting the six TMs are in the lumen; for POM121, only the N-terminal 31 residues reside in the lumen. TMEM33 has six predicted TMs and is the vertebrate homologue of yeast Pom33 but has not yet been experimentally confirmed as a Nup. **c**, The cryo-ET density of Pom152 (the yeast homolog of GP210), colored yellow here, is placed into the reconstruction for one LR protomer (colored grey). Pom152 is thought to contain nine Ig-like domains[1].

**Reference**

1 Upla, P. *et al*. Molecular Architecture of the Major Membrane Ring Component of the Nuclear Pore Complex. *Structure* **25**, 434-445, doi:10.1016/j.str.2017.01.006 (2017).
