# Supplementary material for: Molecular architecture of the luminal ring of the Xenopus laevis nuclear pore complex
Source: Cell Res. 2020 May 4;30(6):532–40. doi: 10.1038/s41422-020-0320-y (PMC7264284; doi:10.1038/s41422-020-0320-y)
Supplement: Supplementary file 12 — Supplementary Table S1 [file 41422_2020_320_MOESM12_ESM.pdf]

**Supplementary information, Table S1 |** Cryo-ET data collection and STA reconstruction statistics.

| | | | | |
|---|---|---|---|---|
| **Data collection** | | | | |
| EM equipment | Titan Krios | | | |
| Voltage (kV) | 300 | | | |
| Detector | Gatan K2 | | | |
| Energy filter | Gatan GIF Quantum, 20 eV slit | | | |
| Pixel size (Å) | 2.222 | | | |
| Tilt schemes | Dose-symmetric scheme | Bi-directional scheme | Continuous scheme | |
| Number of tilt-series | 240 | 874 | 311 | |
| Electron dose ($e^-$/Å$^2$) | 90 ~ 150 | | | |
| Defocus range (μm) | -2.0 ~ -4.0 | | | |
| Software | SerialEM | | | |
| **Reconstruction** | | | | |
| Software | Dynamo/RELION3.0-beta | | | |
| Data set | LR | CR | IR | NR |
| Number of used Particles | 34,087 | 112,196 | 34,086 | 34,068 |
| Final Resolution (Å) | 15.1 | 9.1 | 13.1 | 13.6 |
| Map sharpening B-factor (Å$^2$) | -2000 | -1000 | -2000 | -2000 |
| EMDB accession code | EMD-0983 | EMD-0986 | EMD-0997 | EMD-0998 |
