# Supplementary material for: Molecular architecture of the luminal ring of the Xenopus laevis nuclear pore complex
Source: Cell Res. 2020 May 4;30(6):532–40. doi: 10.1038/s41422-020-0320-y (PMC7264284; doi:10.1038/s41422-020-0320-y)
Supplement: Supplementary file 13 — Supplementary Table S2 [file 41422_2020_320_MOESM13_ESM.pdf]

Supplementary information, Table S2 | Cryo-EM data collection and SPA reconstruction statistics.

**Data collection**				
EM equipment	Titan Krios			
Voltage (kV)	300			
Detector	Gatan K2			
Energy filter	Gatan GIF Quantum, 20 eV slit			
Pixel size (Å)	2.222			
Magnification	22,502			
Data set	Tilt0	Tilt30	Tilt45	Tilt55
Exposure rate ($e^-/(s·Å^2)$)	2.612	2.612	2.612	2.612
Number of frames	40	46	56	70
Total Electron exposure ($e^-/Å^2$)	52	60	78	91
Defocus range (μm)	-1.0 ~ -4.0			
Total Number of images	4,606	4,186	4,158	10,111
Selected Number of images	3,250	2,888	2,745	3,516
Software	AutoEMation2			
**Reconstruction**				
Software	RELION2.1/RELION3.0-beta/THUNDER			
Number of used Particles	311,240			
Symmetry/Final resolution (Å)	C1/10.7			
Masked regions	Grid domain & Finger domain			
Number of Micrographs used	12,399			
Number of extracted particles	612,814			
Final number of particles	311,240			
Local Resolution Ranges (Å)	9 ~ 13			
Map sharpening B-factor ($Å^2$)	-2000			
Accuracy of rotation (˚)	4.48			
Accuracy of translation (pixels)	1.34			
EMDB accession code	EMD-0982			
