# Supplementary material for: Molecular architecture of the luminal ring of the Xenopus laevis nuclear pore complex
Source: Cell Res. 2020 May 4;30(6):532–40. doi: 10.1038/s41422-020-0320-y (PMC7264284; doi:10.1038/s41422-020-0320-y)
Supplement: Supplementary file 14 — Supplementary Video S1 legend [file 41422_2020_320_MOESM14_ESM.pdf]

Supplementary information, Video S1 | Visualization of macromolecular complexes across the NE on a representative tomogram. Reconstructions of the CR, IR, NR, LR and ribosomal subunits were individually projected back into a representative tomogram (low-passed to 80 Å) containing a piece of 824×824 nm nuclear envelope, which is viewed from three distinct angles. Ribosomes: 40S: Small ribosome subunit; 60S: Large ribosome subunit; TRAP: translocon-associated protein complex; OST: oligosaccharyl-transferase.
